# Supplementary figures and images for: The human olfactory bulb communicates perceived odor valence to the piriform cortex in the gamma band and receives a refined representation back in the beta band
Source: PLoS Biol. 2024 Oct 14;22(10):e3002849. doi: 10.1371/journal.pbio.3002849 (PMC11501019; doi:10.1371/journal.pbio.3002849)

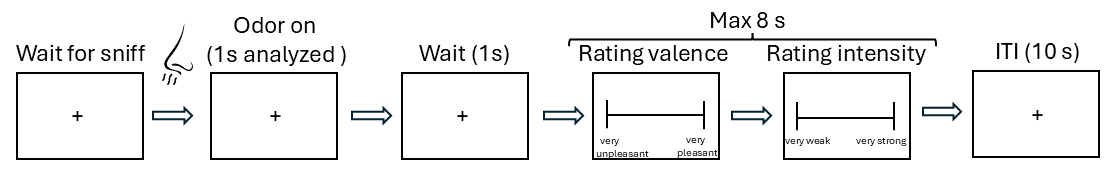

Supplement: S1 Fig — The trial starts when a sniff is detected (marked by the nose), after which a pre-set order of events are triggered. Values in the figure are seconds (s) and arrows represent direction of events. A max limit of 8 s is used for rating and is followed by a minimum inter-trial-interval (ITI) of 10 s to decrease odor habituation. Note that there was no time limit for the “Wait for sniff” event meaning that the total ITI was longer than 10 s in nearly all trials. Moreover, odor length differs between experiments to control for potential odor offset effect. (PNG) [file pbio.3002849.s001.png]
